# Supplementary material for: Genomic and Functional Regulation of TRIB1 Contributes to Prostate Cancer Pathogenesis
Source: Cancers (Basel). 2020 Sep 11;12(9):2593. doi: 10.3390/cancers12092593 (PMC7565426; doi:10.3390/cancers12092593)
Supplement: Supplementary file 1 [file cancers-12-02593-s001.pdf]

Supplementary Materials: Genomic and functional regulation of TRIB1 contributes to prostate cancer pathogenesis

Parastoo Shahrouzi, Ianire Astobiza, Ana R Cortazar, Verónica Torrano, Alice Macchia, Juana M. Flores, Chiara Niespolo, Isabel Mendizabal, Ruben Fernandez-Caloto, Amaia Ercilla, Laura Camacho, Leire Arreal, Maider Bizkarguenaga, Maria L. Martinez-Chantar, Xose R. Bustelo, Edurne Berra, Endre Kiss-Toth, Guillermo Velasco, Amaia Zabala-Letona, Arkaitz Carracedo and TRAIN Consortium

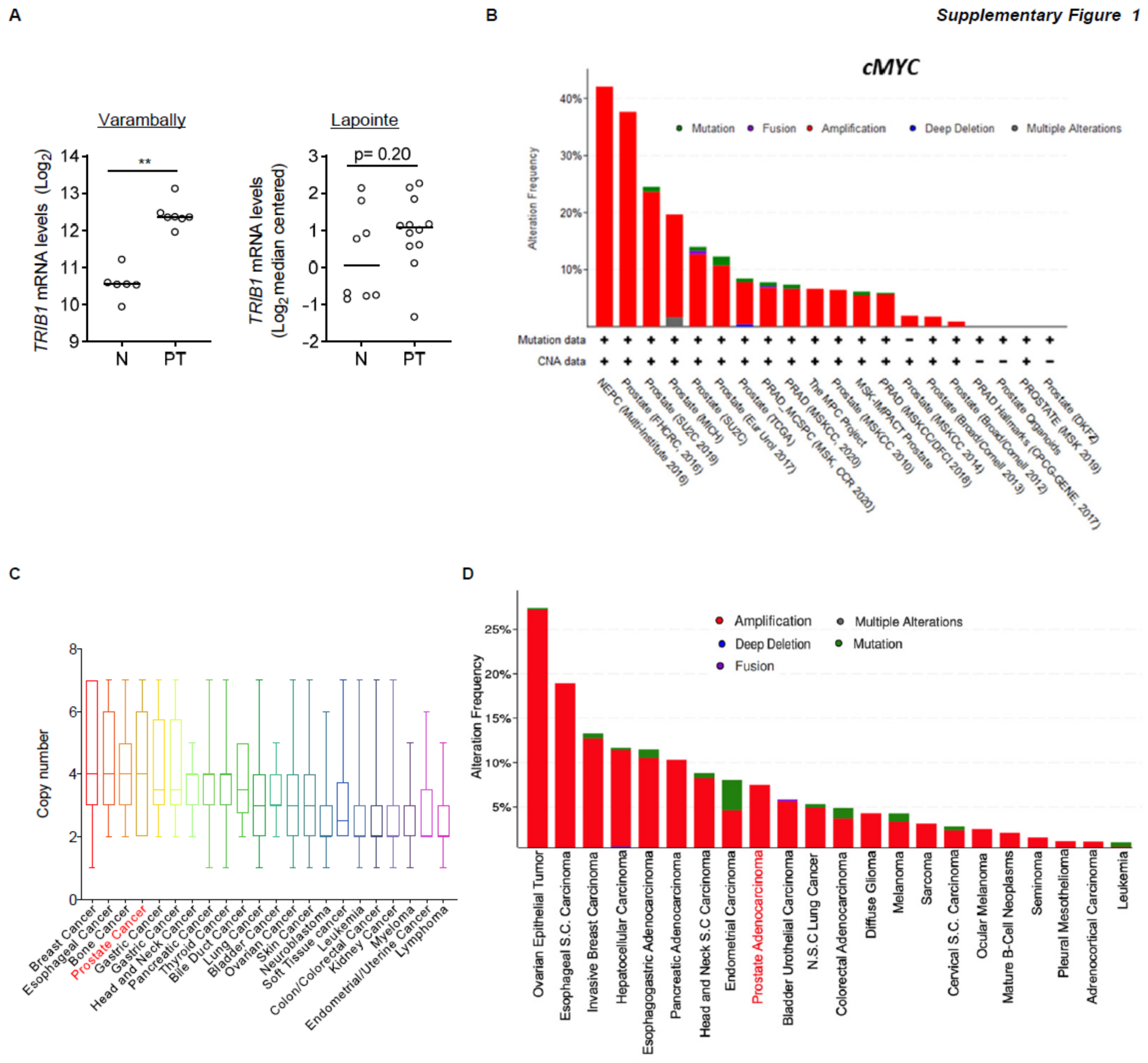

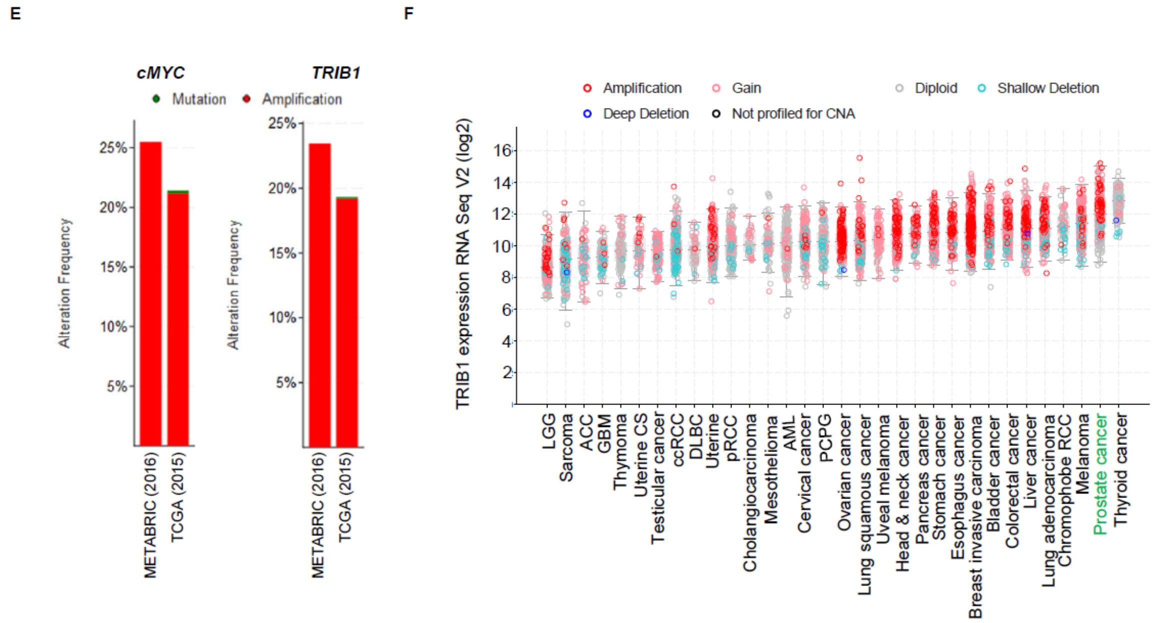

Figure S1. *TRIB1* is amplified and overexpressed in cancers. (**A**) Gene expression analysis of *TRIB1* in two human prostate cancer datasets in normal (N) versus primary tumors (PT). Data extracted from Cancertool. Each dot indicates one individual. *, $p < 0.05$. **, $p < 0.01$. ***, $p < 0.001$. Statistics: Two-tailed Mann-Whitney U test. (**B**) Genomic alterations of *cMYC* in the indicated prostate cancer datasets. Plot extracted from cBioportal. (**C**) Copy Number Alteration analysis of several cell lines across multiple cancer type. Data extracted from DepMap Portal (Broad Institute). (**D**) Genomic alterations of *TRIB1* in the indicated TCGA cancer datasets. Plot extracted from cBioportal. (**E**) Genomic alterations of *TRIB1* in the indicated breast cancer datasets. Plot extracted from cBioportal. (**F**) *TRIB1* gene expression levels in TCGA cancer datasets, with individual annotation of copy number alterations (colored circles). Data extracted from cBioportal.

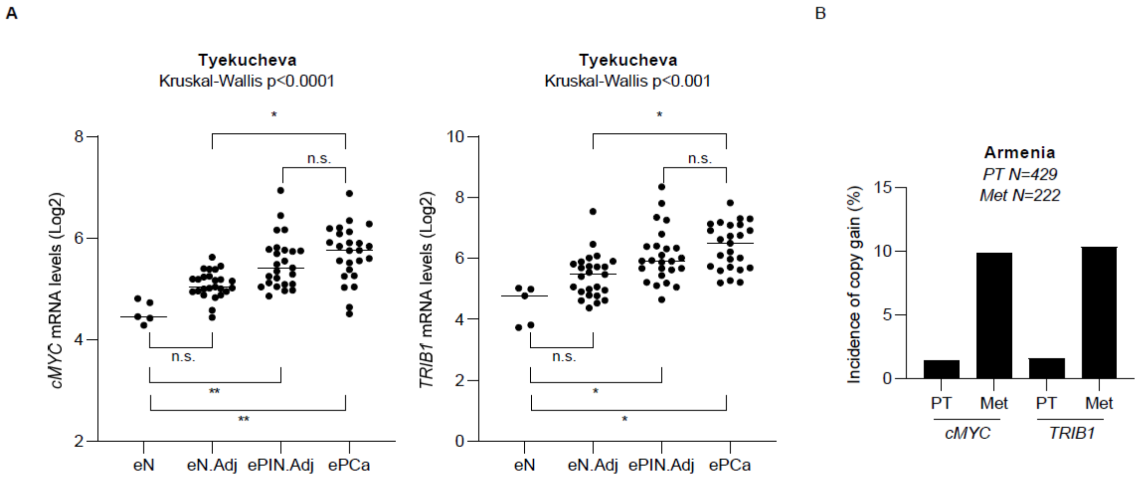

C

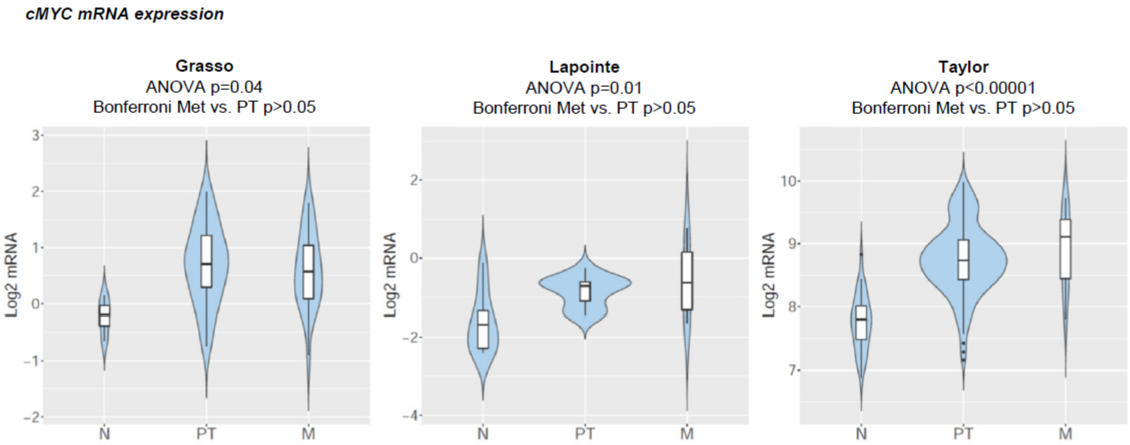

D

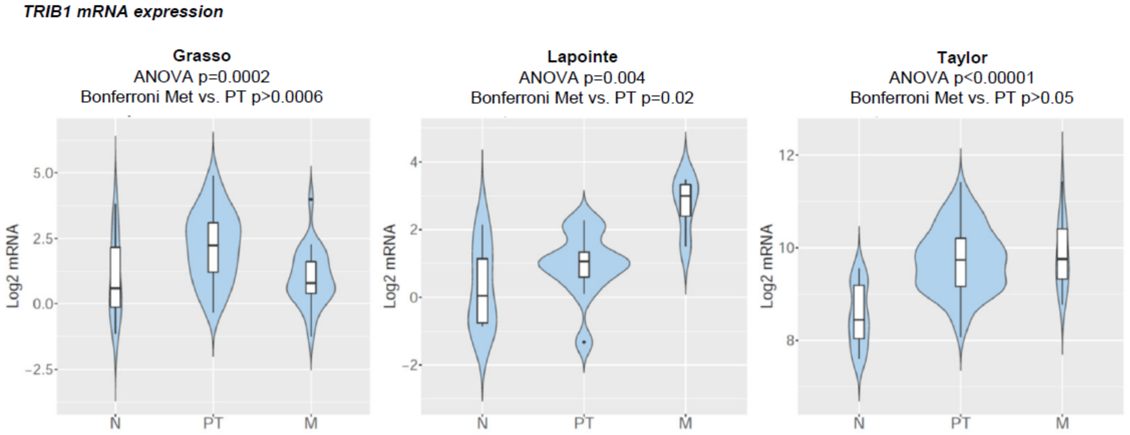

Figure S2. Association of *cMYC* and *TRIB1* genomic and transcriptional alterations to distinct prostate cancer pathological scenarios. (**A**) mRNA expression of *TRIB1* and *cMYC* in (Tyekucheva S, Bowden M, Bango C, et al. Nat Commun. 2017;8(1):420). The expression in the epithelial compartment of the indicated pathological scenarios is indicated. eN: epithelial tissue from normal prostate; eN.Adj: epithelial tissue from normal prostate tissue region adjacent to prostate cancer; ePIN.Adj: epithelial tissue from prostate intraepithelial neoplasia region adjacent to prostate cancer; ePCa: epithelial tissue from prostate cancer tissue. Statistics: Kruskal-Wallis with Dunn´s multiple testing. *, $p < 0.05$; **, $p < 0.01$. (**B**) Frequency of *cMYC* and *TRIB1* copy gain in tissue from localized prostate cancer or metastatic disease in the study (Armenia J, Wankowicz SAM, Liu D, et al. The long tail of oncogenic drivers in prostate cancer. Nat Genet. 2018;50(5):645-651). (**C-D**) mRNA expression of *cMYC* (**C**) and *TRIB1* (**D**) in non-tumoral specimens (N), primary tumor specimens (PT) and metastatic lesions (Met) in the indicated datasets. Data extracted from Cancertool.

***TRIB1* expression**

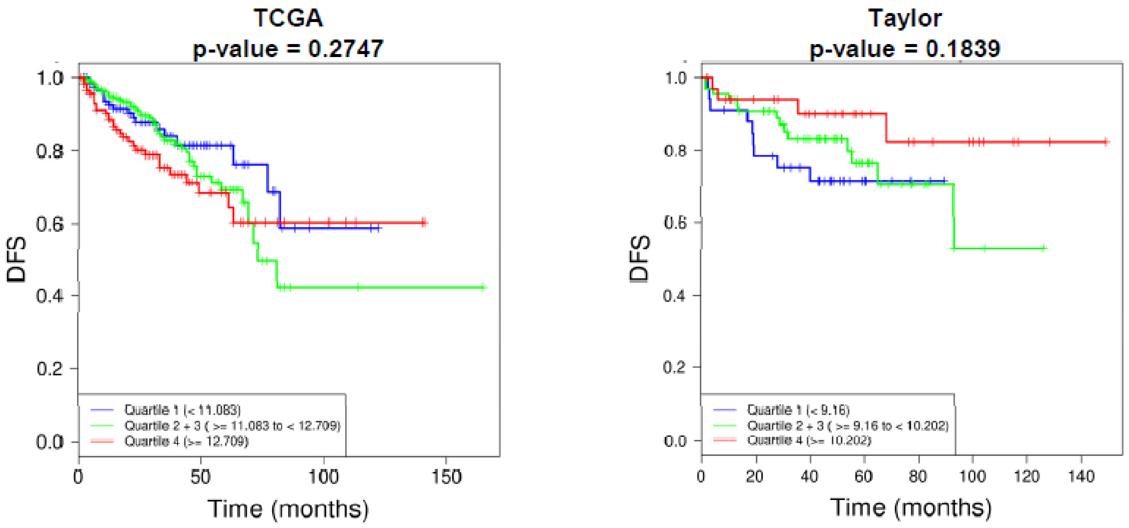

***cMYC* expression**

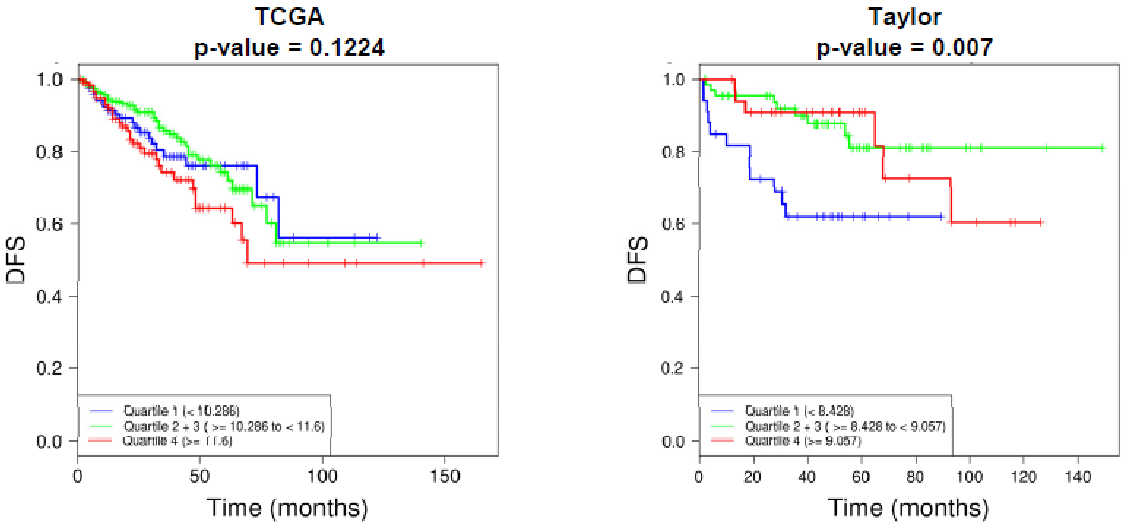

cMYC-TRIB1 **signature (average expression per patient)**

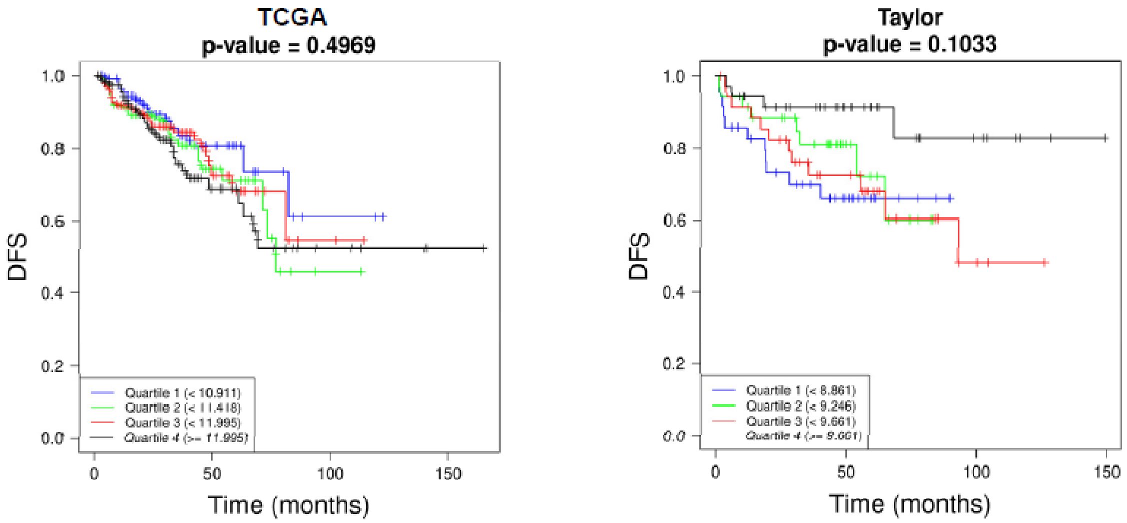

Figure S3. Association of *cMYC* and *TRIB1* mRNA levels with biochemical recurrence in prostate cancer. Association to biochemical recurrence upon prostatectomy (Disease-free survival, DFS) of *TRIB1* expression (top), *cMYC* expression (middle) or the average signal computed from both genes (bottom). Kaplan-Meyer representations are included and Kaplan-Meyer estimator was used to calculate the p-value. Patients were divided according to the quartiles of expression of the indicated gene or gene combination.

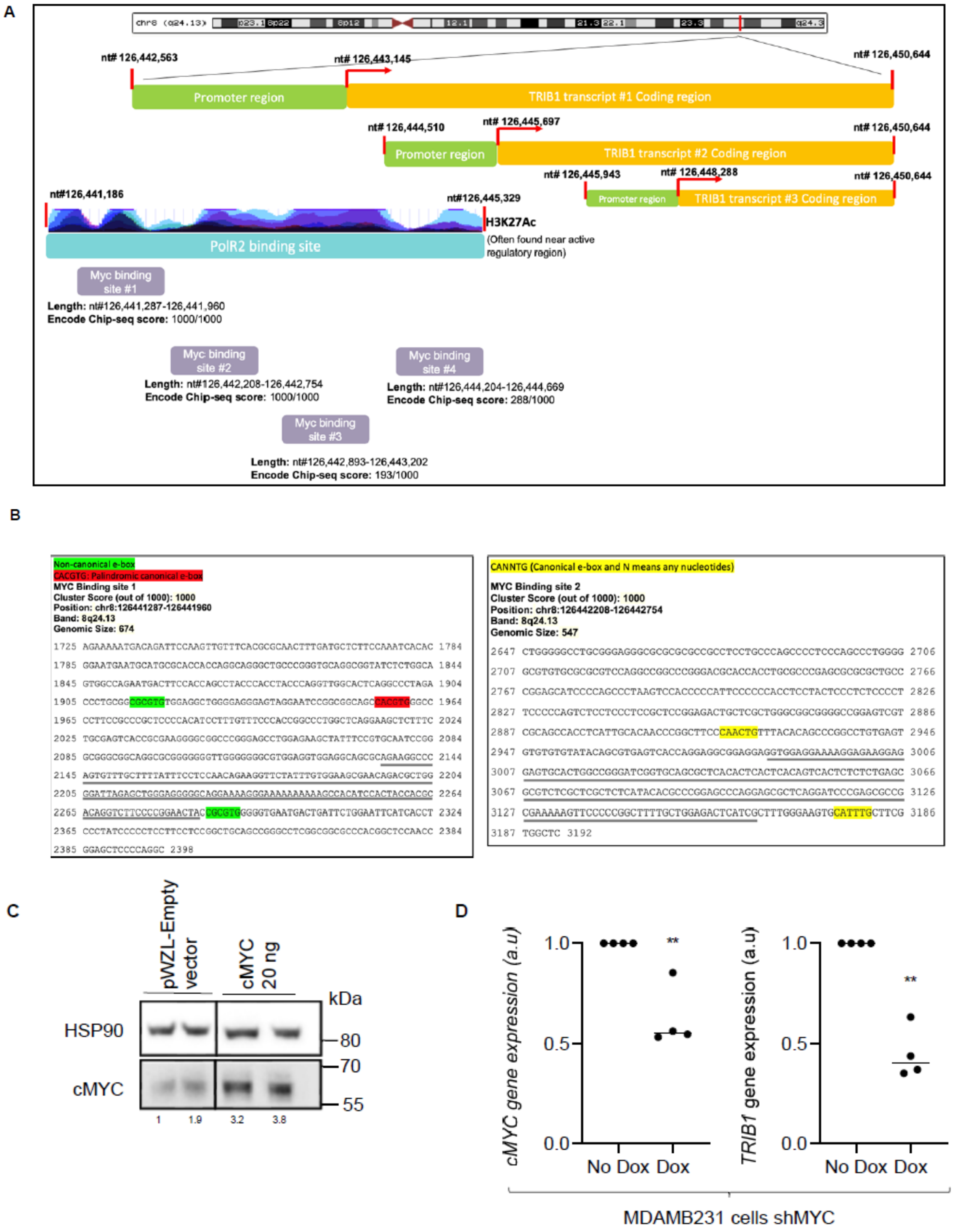

Figure S4. Transcriptional regulation of *TRIB1* by cMYC. (**A**) Schematic demonstration of the regulatory region of three transcripts of *TRIB1* on chromosome 8q24. cMYC binding sites on *TRIB1* regulatory region is shown in purple boxes with the nucleotide length of the site based on ChIP-Seq data from ENCODE project (https://genome.ucsc.edu). nt: nucleotide; Green: Promoter region; Yellow: coding region; Blue: PolR2A binding site on *TRIB1* regulatory region. red lines: Start and end of the coding region, start of the promoter region. Coordinates represent the human assembly hg19. (**B**) Illustration of the sequences of two cMYC binding regions, with the predicted amplicon in the ChIP-RTqPCR assay (grey lines) and the location of putative cMYC binding sites. Colors indicate the type of binding sites. (**C**) Evaluation of the protein levels of cMYC by western blot following transient overexpression of cMYC in HEK293FT cells for 24 hours. HSP90. serves as the housekeeping control. pWZL empty vector serves as the negative control. Western blot is representative of 3 independent

experiments (densitometry of cMYC relative to HSP90 is indicated). (**D**) Impact of inducible cMYC silencing on *TRIB1* mRNA expression in MDAMB231 breast cancer cells. Left panel shows cMYC downregulation upon activation of the shRNA with 150 ng/ml of doxycycline for 6 days, and right panels depict *TRIB1* mRNA abundance (values are normalized to no dox and relative to *GAPDH* mRNA abundance). a.u: arbitrary unit. Statistics: One Sample t-test. *, $p < 0.05$; **, $p < 0.01$.

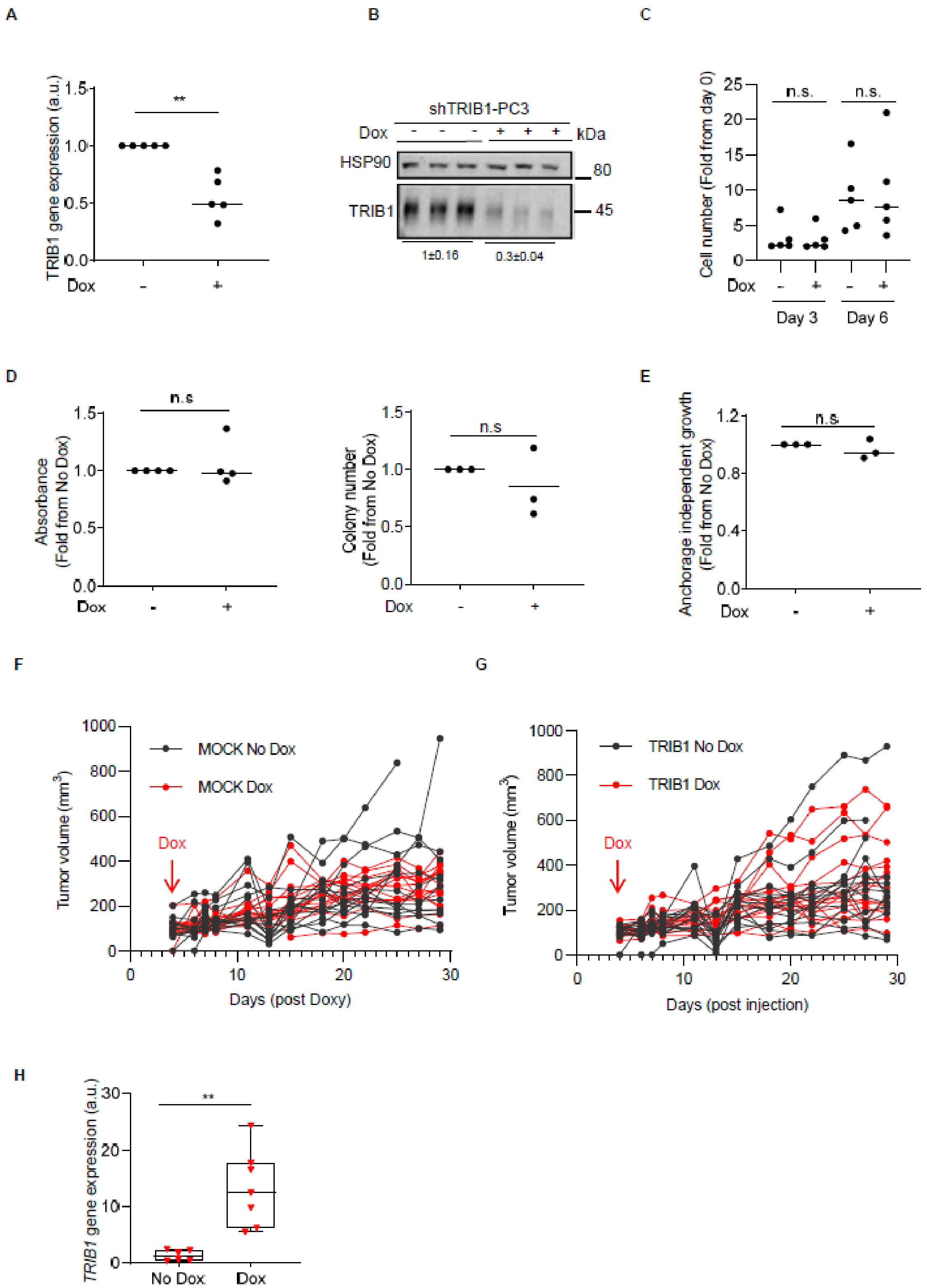

Figure S5. TRIB1 silencing in PC3 cells is inconsequential for tumor cell function. Validation of TRIB1 silencing in PC3 by RTqPCR (**A**) and protein expression (**B**). Each dot in (**A**) represents one biological replicate. HSP90 serves as a housekeeping control for western blot analysis (densitometry of TRIB1 relative to HSP90 is indicated, mean ± standard error). *β-ACTIN* was used for normalization in RTqPCR analysis. 200 ng/ml doxycycline was used to induce the expression of shRNA. RTqPCR values were normalized to the expression of non-induced cells. a.u: arbitrary unit. Statistics: One sample student ttest. **, p<0.01. (**C**) PC3 cell growth was measured by crystal violet staining at day 0, and after 3- or 6-days post-doxycycline induction. Each dot represents one biological replicate. ns:

statistically not significant a.u: arbitrary unit. Statistics: Paired student t-test. (**D**) Evaluation of the effect of TRIB1 silencing on the clonal growth. Colonies formed by PC3 cells were counted and the crystal violet absorbance was measured after 21 days (Left and central panels). Each dot represents one biological replicate. ns: statistically not significant a.u: arbitrary unit. Statistics: One sample student t-test. (**E**) Analysis of the anchorage independent growth of PC3 cells upon silencing of TRIB1. Colonies were counted 3 weeks after seeding. Each dot represents one biological replicate. ns: statistically not significant a.u: arbitrary unit. Statistics: One sample student t-test. (**F-H**) Impact of inducible TRIB1 ectopic expression on the growth of DU145 cells *in vivo* in the flank of immunocompromised mice. Tumor volume is illustrated for DU145 cells transduced with MOCK (**F**) or inducible TRIB1 lentivirus (**G**). A control of the induction by doxycycline *in vivo* is shown in H. Statistics in H: Student T-test.

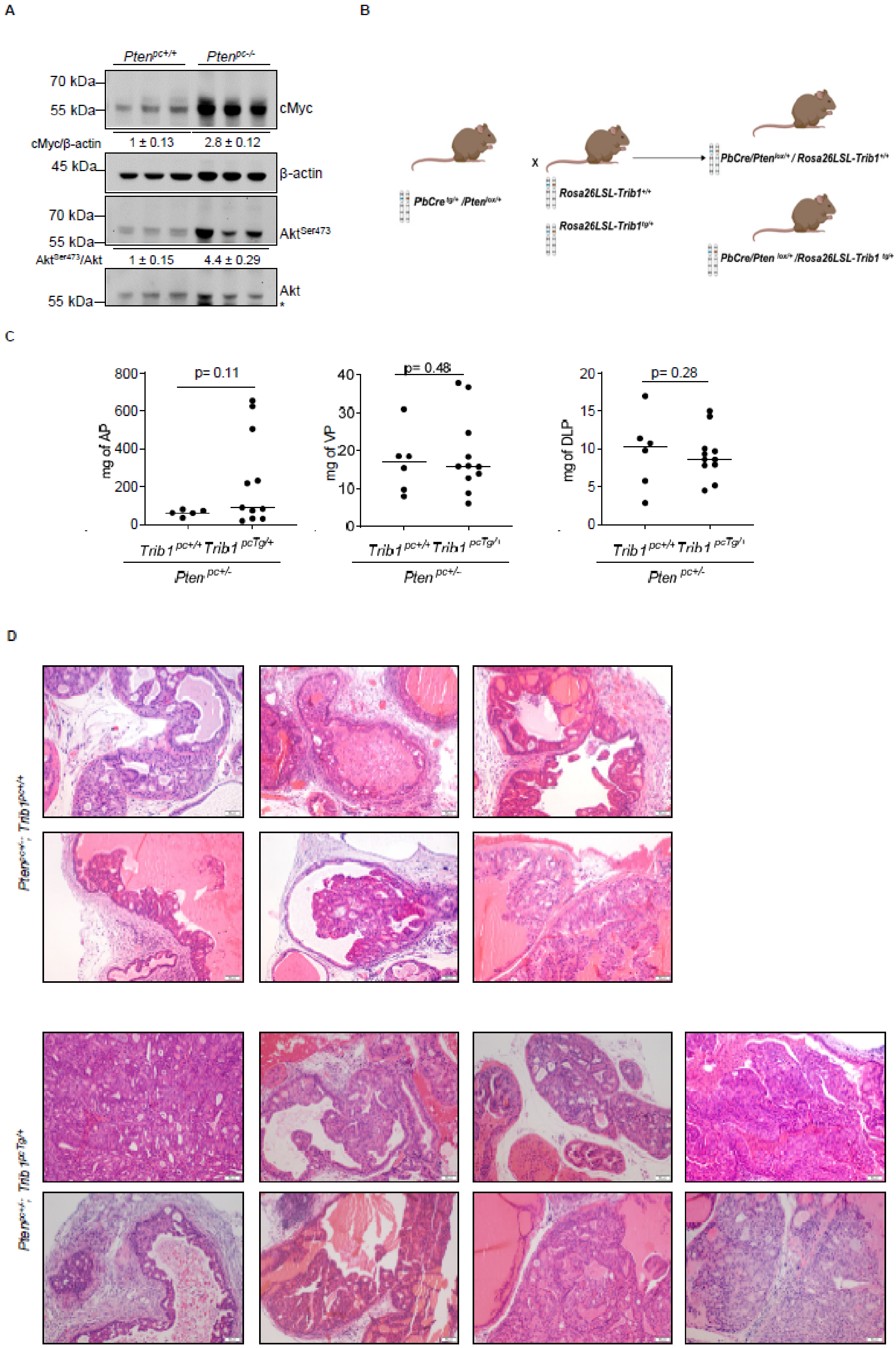

Figure S6. Prostate-specific TRIB1 transgenic expression in mice. (**A**) Representative western blot of cMyc protein abundance in prostate tissue from 6-monthold *Pten*$^{pc+/+}$ (n=3) and *Pten*$^{pc-/-}$ mice (n=3) (densitometry of cMyc relative to HSP90 and p-Akt relative to Akt is indicated, mean ± standard error). (**B**) Schematic illustration of the generation of prostate-specific *Trib1* transgenic mouse models of

prostate cancer. Mice with conditional deletion of Pten in prostate (Pb-Cre4-Pten +/-) were crossed with mice carrying a conditional transgenic *Trib1* allele (Rosa26LSL-Trib1Tg/+). Pb: probasin. Allelic changes: +: Wildtype allele; -: deleted allele; Tg: transgenic allele. (**C**) Analysis of anterior (AP), ventral prostate (VP) and dorsolateral prostate (DLP) mass (mg) in 15-17-month-old Ptenpc+/- /Trib1pc+/+ (n=5-6) and Ptenpc+/-/Trib1pcTg/+ (n=11) mice. Statistics: Two-tailed Mann-Whitney U test. (**D**) Extended presentation of representative hematoxylin-eosin images for *Pten*$^{pc+/-}$ *Trib1*$^{pc+/+}$ (N=6) and *Pten*$^{pc+/-}$; *Trib1*$^{pcTg/+}$ (N=8) mice from figure 4D.

Figure 2E

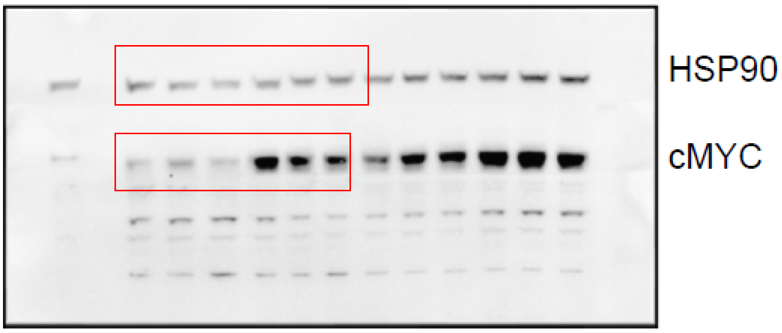

Figure 3B

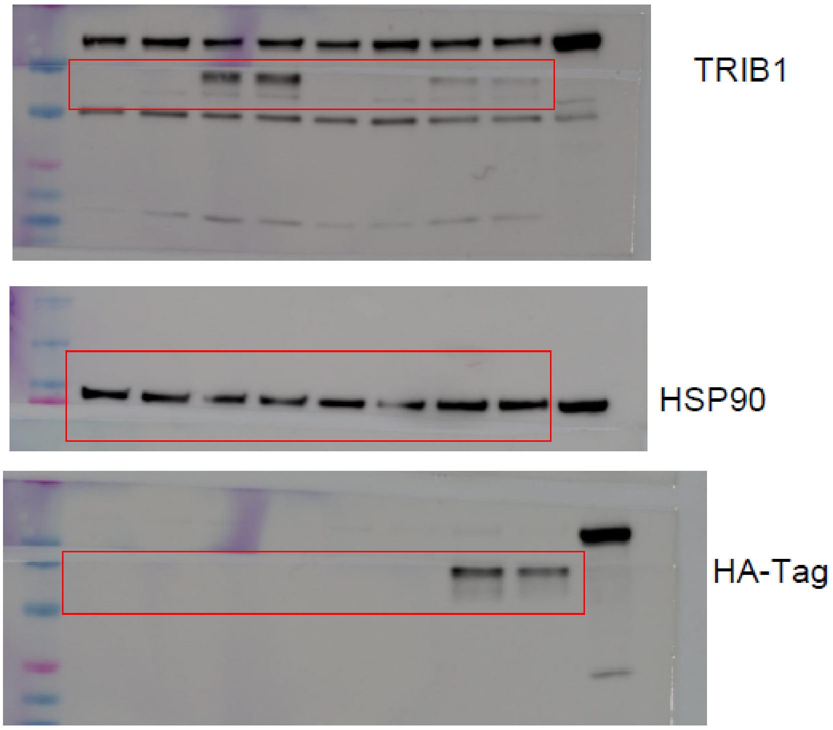

Figure 4A

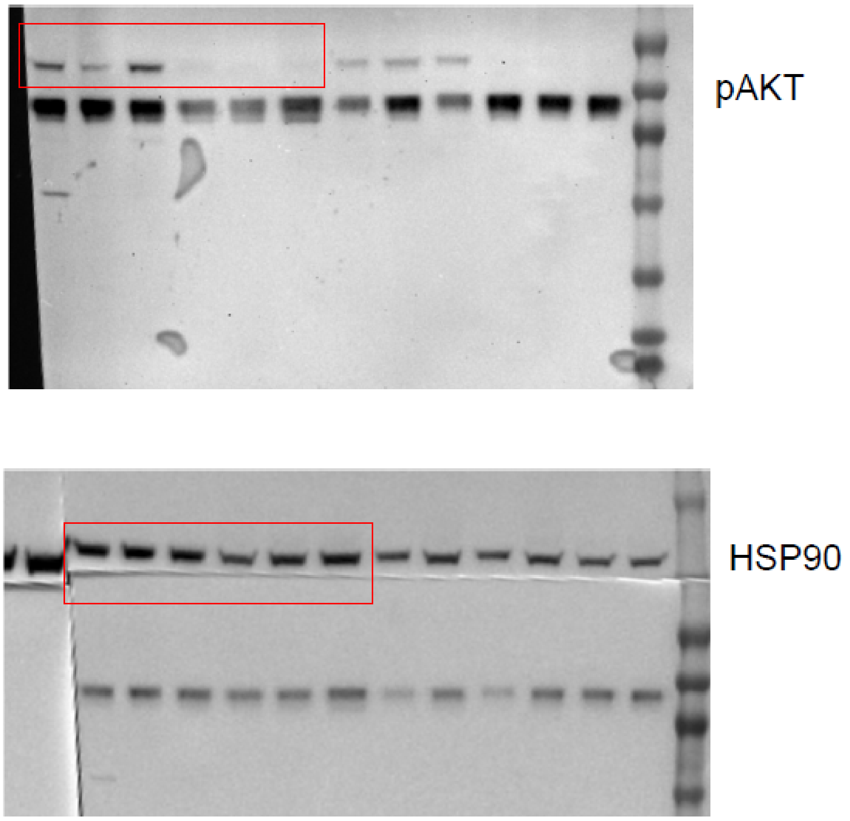

Figure S4C

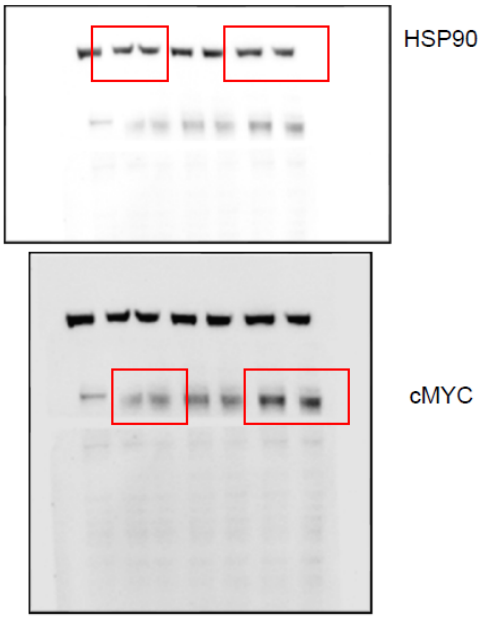

Figure S5B

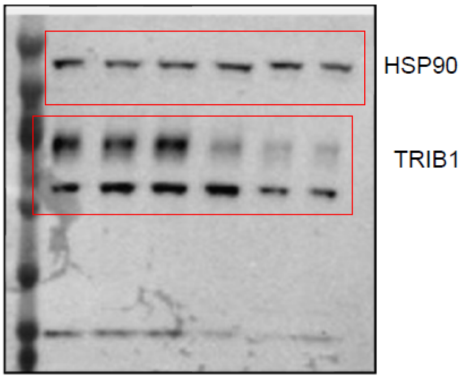

Figure S6A

Figure S7. Uncropped western blot figures.

Table S1. qPCR and UPL probes used in this study.

Gene	Species	Technology	Forward primer sequence	Reverse primer sequence	Probe number (according to Roche codes)
TRIB1	HUMAN	UPL Roche	cctgaagcttaggaagttcgtc	gatctcagggctcacgtagg	46
TRIB1	HUMAN	UPL Roche	cggctcttcaagcagattgt	gactttctagtctaactgggttctc	87
TRIB1	MOUSE	UPL Roche	ctagctgagcgcgagcat	tgccagtgatgttgctatgg	42
TRIB1	MOUSE	UPL Roche	cctgaagctcaggaagttcg	ccaggctttccagtctaagc	91
GAPDH	HUMAN	Taqman/SYBR	Hs02758991-g1	----	----
GAPDH	MOUSE	Taqman/SYBR	Mm99999915_g1	----	----
c-MYC	HUMAN	UPL Roche	gctgcttagacgctggattt	taacgttgaggggcatcg	66
ß-Actin	HUMAN	Taqman/SYBR	Hs99999903-m1	----	----

Table S2. Antibodes used in this study.

Antibody	References	Host specie	Species reactivity
**TRIB1**	**MerckMillipore #09-126**	**Rabbit**	**Human**
**HSP90**	**Cell signaling technology #4874**	**Rabbit**	**Mouse/Human**
**cMYC**	**Cell signaling technology #5605**	**Rabbit**	**Mouse/Human**
**pAKT^{S473}**	**Cell signaling technology #4060**	**Rabbit**	**Mouse/Human**
**F4/80**	**BIORAD**	**Rat**	**Mouse**
**HA-Tag**	**Covance #16B12**	**Mouse**	**Human**
**Secondary AB**	**Jackson immune research**	**Mouse**	**Mouse/Human**
**Secondary AB**	**Jackson immune research**	**Rabbit**	**Mouse/Human**
**Secondary AB**	**Jackson immune research**	**Mouse**	**Mouse/Human**
**ki67**	**Ventana #790-4286**	**Rabbit**	**Mouse/Human**

Table S3. Primers employed in ChIP analysis.

Name	Sequence
MYC-Binding site #1:primer Fw	AGAAGGCCCAGTGTTTGCT
MYC-Binding site #1:primer Rv	GTAGTTCCGGGGAAGACCTG
MYC-Binding site #2:primer Fw	GGTGGAGGAAAAGGAGAAGG
MYC-Binding site #2:primer Rw	CGATGAGTCTCCAGCAAAAG

Table S4. Genes in peak in *cMYC* amplicon n different tumour types.

Cancer Subset	In Peak?	Nearest Peak	#Genes in Peak	Q-Value
Epithelial cancers	Yes	chr8:128740968-128762864	1	0.0
Ovarian serous cystadenocarcinoma	Yes	chr8:128494666-129683434	11	1.79E-148
Breast invasive adenocarcinoma	Yes	chr8:128668369-128777531	1	5.83E-78
Lung cancers	Yes	chr8:128709604-128777531	1	5.21E-49
Uterine corpus endometrioid carcinoma	Yes	chr8:128740968-128762864	1	3.91E-34
Colorectal cancers	Yes	chr8:128217188-128754563	3	4.25E-24
Colon adenocarcinoma	Yes	chr8:128390660-128754563	3	4.4E-17
Brain lower grade glioma	Yes	chr8:117413831-135052769	91	8.39E-16
Glial cancers	Yes	chr8:127927969-131296551	19	3.12E-12
Bladder urothelial carcinoma	Yes	chr8:127579104-129787426	14	8.3E-11
Liver hepatocellular carcinoma	Yes	chr8:113199019-145232496	224	1.21E-6
Uterine carcinosarcoma	Yes	chr8:128630521-129099256	9	4.46E-6
Pancreatic adenocarcinoma	Yes	chr8:127865119-128800301	4	3.21E-4
Cutaneous melanoma	Yes	chr8:101237993-145232496	271	0.00702
Glioblastoma multiforme	Yes	chr8:127927969-131798497	21	0.0379
Prostate adenocarcinoma	Yes	chr8:119897767-129710968	60	0.0619
Blood cancers	Yes	chr8:111029607-145232496	224	0.186

Table S5. mRNA expression values for the indicated genes in *cMYC* amplicon (primary tumours vs. Normal prostate specimens). Negative fold change indicates downregulation in tumours vs. Primary tissue. For details on the calculation, see methods.

GeneSymbol	Grasso.logRatio	Grasso.Directional.FoldChange	Grasso.p.value	Lapointe.logRatio	Lapointe.Directional.FoldChange	Lapointe.p.value	Taylor.logRatio	Taylor.Directional.FoldChange	Taylor.p.value	Tomlins.logRatio	Tomlins.Directional.FoldChange	Tomlins.p.value	Varambally.logRatio	Varambally.Directional.FoldChange	Varambally.p.value	AverageFC
FBXO32	-0.681	-1.603	0.000	-0.527	-1.441	0.059	-0.600	-1.516	0.000	-0.185	-1.137	0.016	-0.513	-1.427	0.018	-1.425
DEPTOR	0.022	1.015	0.890	NA	NA	NA	-0.555	-1.469	0.000	NA	NA	NA	-0.893	-1.857	0.001	-0.770
MTSS1	-0.431	-1.348	0.000	-0.300	-1.231	0.049	-0.225	-1.169	0.000	-0.093	-1.067	0.257	-0.098	-1.070	0.475	-1.177
WDYHV1	0.295	1.227	0.037	NA	NA	NA	0.122	1.088	0.028	NA	NA	NA	-0.228	-1.171	0.113	0.381
FAM91A1	0.543	1.457	0.004	-0.136	-1.099	0.479	0.266	1.203	0.002	0.160	1.118	0.027	0.168	1.123	0.290	0.760
FAM84B	0.059	1.042	0.778	0.660	1.580	0.023	0.173	1.127	0.000	NA	NA	NA	0.387	1.308	0.033	1.264
PVT1	0.194	1.144	0.200	0.846	1.797	0.000	NA	NA	NA	0.391	1.311	0.000	0.839	1.789	0.000	1.510
MAL2	0.836	1.785	0.002	1.070	2.100	0.009	0.580	1.495	0.000	0.345	1.270	0.000	0.644	1.562	0.035	1.642
MYC	0.917	1.888	0.003	0.784	1.721	0.018	0.996	1.995	0.000	0.267	1.203	0.001	1.467	2.765	0.000	1.915
TRIB1	1.145	2.212	0.034	0.644	1.563	0.238	1.190	2.282	0.000	NA	NA	NA	1.859	3.627	0.000	2.421
TRMT12	0.291	1.224	0.003	0.644	1.343	0.021	0.147	1.108	0.003	-0.134	-1.097	0.004	-0.172	-1.127	0.349	0.290
SQLE	-0.913	-1.883	0.001	NA	NA	NA	-0.195	-1.145	0.094	0.123	1.089	0.252	-1.021	-2.029	0.001	-0.992
ENPP2	-0.545	-1.459	0.015	-0.279	-1.213	0.417	-0.237	-1.178	0.035	-0.132	-1.096	0.117	-0.103	-1.074	0.739	-1.204
COL14A1	0.242	1.183	0.443	-0.862	-1.817	0.008	-0.166	-1.122	0.242	-0.316	-1.245	0.020	-0.131	-1.095	0.815	-0.819
ZNF572	0.111	1.080	0.477	NA	NA	NA	-0.135	-1.098	0.005	NA	NA	NA	-0.540	-1.454	0.098	-0.491
HAS2	0.755	1.687	0.101	-0.745	-1.677	0.012	-0.077	-1.055	0.292	NA	NA	NA	-0.441	-1.357	0.052	-0.600
ATAD2	-0.164	-1.120	0.257	-0.272	-1.207	0.022	0.061	1.043	0.439	0.234	1.176	0.137	-0.377	-1.299	0.054	-0.282
C8ORF76	-0.296	-1.228	0.106	-0.071	-1.051	0.548	-0.016	-1.011	0.592	NA	NA	NA	0.120	1.086	0.519	-0.551
ZHX2	0.016	1.011	0.932	-0.073	-1.052	0.680	0.008	1.006	0.890	NA	NA	NA	-0.192	-1.143	0.138	-0.044
ZHX1	0.203	1.151	0.478	-0.336	-1.263	0.016	-0.192	-1.142	0.001	0.109	1.078	0.247	-0.064	-1.046	0.619	-0.244
TMEM65	0.085	1.061	0.716	-0.155	-1.114	0.481	-0.106	-1.076	0.003	-0.056	-1.039	0.389	0.115	1.083	0.560	-0.217
POU5F1B	NA	NA	NA	NA	NA	NA	NA	NA	NA	NA	NA	NA	-0.017	-1.012	0.915	-1.012
HAS2-AS1	NA	NA	NA	NA	NA	NA	NA	NA	NA	-0.010	-1.007	0.900	NA	NA	NA	-1.007

FAM83A	-0.332	-1.259	0.258	-0.001	-1.001	0.993	0.077	1.055	0.218	-0.069	-1.049	0.146	0.393	1.313	0.266	-0.188
NDUFB9	-0.138	-1.100	0.033	NA	NA	NA	0.117	1.084	0.077	NA	NA	NA	0.066	1.046	0.372	0.344
DERL1	0.071	1.050	0.511	-0.128	-1.093	0.301	0.116	1.084	0.110	0.007	1.005	0.905	0.064	1.045	0.660	0.618
RNF139	-0.144	-1.105	0.245	-0.008	-1.005	0.956	0.009	1.007	0.801	0.234	1.176	0.002	0.057	1.040	0.479	0.223
FER1L6	NA	NA	NA	NA	NA	NA	0.039	1.027	0.571	NA	NA	NA	NA	NA	NA	1.027
TAF2	0.128	1.093	0.041	0.115	1.083	0.185	0.075	1.053	0.306	-0.104	-1.075	0.041	0.015	1.010	0.833	0.633
ANXA13	0.304	1.234	0.277	NA	NA	NA	0.089	1.064	0.209	-0.136	-1.099	0.064	0.036	1.025	0.955	0.556
KLHL38	NA	NA	NA	NA	NA	NA	0.085	1.061	0.121	NA	NA	NA	NA	NA	NA	1.061
SNTB1	0.852	1.806	0.025	-0.214	-1.160	0.413	-0.086	-1.061	0.086	0.009	1.006	0.855	-0.064	-1.045	0.797	-0.091
MRPL13	0.373	1.295	0.053	0.201	1.149	0.313	-0.015	-1.010	0.611	NA	NA	NA	0.058	1.041	0.795	0.619
TNFRSF11B	0.064	1.046	0.771	0.345	1.270	0.291	0.077	1.055	0.112	0.024	1.017	0.826	0.386	1.307	0.336	1.139
NSMCE2	0.660	1.580	0.001	-0.091	-1.065	0.427	0.164	1.120	0.031	0.042	1.030	0.317	0.180	1.133	0.115	0.760
TATDN1	0.558	1.473	0.000	NA	NA	NA	0.048	1.034	0.489	NA	NA	NA	0.032	1.022	0.816	1.176
MTBP	0.298	1.229	0.422	0.478	1.393	0.287	0.049	1.035	0.052	NA	NA	NA	0.034	1.024	0.891	1.170
FER1L6-AS1	-0.052	-1.037	0.864	NA	NA	NA	NA	NA	NA	NA	NA	NA	0.675	1.596	0.400	0.280
DSCC1	0.155	1.113	0.357	NA	NA	NA	0.042	1.030	0.180	NA	NA	NA	0.751	1.683	0.135	1.275
NOV	-0.056	-1.040	0.882	0.166	1.122	0.675	0.034	1.024	0.723	NA	NA	NA	1.653	3.144	0.003	1.062
COLEC10	0.549	1.463	0.473	0.109	1.078	0.742	0.107	1.077	0.108	NA	NA	NA	1.183	2.271	0.187	1.472
MIR1204	NA	NA	NA	NA	NA	NA	NA	NA	NA	NA	NA	NA	NA	NA	NA	NA
MIR1205	NA	NA	NA	NA	NA	NA	NA	NA	NA	NA	NA	NA	NA	NA	NA	NA
MIR1206	NA	NA	NA	NA	NA	NA	NA	NA	NA	NA	NA	NA	NA	NA	NA	NA
MIR1207	NA	NA	NA	NA	NA	NA	NA	NA	NA	NA	NA	NA	NA	NA	NA	NA
MIR1208	NA	NA	NA	NA	NA	NA	NA	NA	NA	NA	NA	NA	NA	NA	NA	NA
MIR4663	NA	NA	NA	NA	NA	NA	NA	NA	NA	NA	NA	NA	NA	NA	NA	NA
PCAT1	NA	NA	NA	NA	NA	NA	NA	NA	NA	NA	NA	NA	NA	NA	NA	NA
ZHX1-C8ORF76	NA	NA	NA	NA	NA	NA	NA	NA	NA	NA	NA	NA	NA	NA	NA	NA

© 2020 by the authors. Submitted for possible open access publication under the terms and conditions of the Creative Commons Attribution (CC BY) license (http://creativecommons.org/licenses/by/4.0/).
